# The effects of stimulation intervention on speech development of children 1-3 years old in Iodine Deficiency Disorders (IDD) area

**DOI:** 10.1186/1687-9856-2013-S1-P148

**Published:** 2013-10-03

**Authors:** Asri Purwanti, Saldi Fitra, Agustini Utari, Rudy Susanto

**Affiliations:** 1 Department of Pediatric, Faculty of Medicine, Diponegoro University/ Dr. Kariadi Hospital, Semarang, Indonesia

**Keywords:** stimulation, speech development, iodine deficiency disorders

## 

Iodine deficiency disorders (IDD) may affect the child growth and development. Speech development disorders in IDD area caused by brain damage. The incidence of speech developmental disorders is still high. Stimulation to children under 3 years old may increase speech capability due to brain plasticity. The aims of this study is to determine the effect of stimulation on speech development of 1-3 years old children in IDD and Non IDD areas.

Quasi experimental study used one group pretest posttest design with consecutive sampling was done on 1- 3 years old children who fulfilled inclusion criteria in Kepil (Non IDD area) and Kertek District (IDD area), Wonosobo regency from April to September 2011. Stimulation interventions in accordance with the guidelines for implementation of stimulation, early detection and intervention for growth and child development in Primary Health Care 2006. Standard score equivalent of global language from Early Language Milestones Scale 2 was measured before and after stimulation in IDD and Non IDD areas. Statistical analysis used paired and independent t test.

Eighty children consisting of 57.5% boys and 43.5% girls were enrolled in this study. Mean of speech development score in IDD 8,4 (SD 7,94) while in Non IDD 2,93 (SD 8,3) with p value 0.004. Standard score equivalent of speech development before and after stimulation in Non IDD area increase from 89.8 to 92.7 (SD 8.3) (p=0.032) and 85.7 to 94 (SD 7.94) (p=0.001) in IDD area. These result suggest that stimulation intervention has an effect on increasing speech development of 1-3 years old children in IDD and Non IDD area.

